# ﻿A new species of *Elpidium* Müller, 1880 (Crustacea, Ostracoda) from Hispaniola, with an updated key to the species of the genus, and its molecular phylogenetic positioning within the Cytheroidea

**DOI:** 10.3897/zookeys.1233.126611

**Published:** 2025-03-28

**Authors:** Francesc Mesquita-Joanes, Ángel Gálvez, Ferran Palero, Juan Rueda

**Affiliations:** 1 Cavanilles Institute of Biodiversity and Evolutionary Biology, University of Valencia, Catedràtic José Beltrán Martinez, 2, 46980 Paterna, Spain University of Valencia Paterna Spain

**Keywords:** Dominican Republic, Limnocytheridae, Neotropical aquatic biodiversity, phytotelmata, Timiriaseviidae

## Abstract

The ostracod genus *Elpidium*, a specialist of phytotelma habitats, has received increased attention during the past decade, with a proliferation of described species, rising from seven to nineteen. These recent studies emphasize the high diversity and endemicity of the genus, and its wide distribution in the Neotropics. Yet many regions are still to be inspected for the presence of *Elpidium*. In this work, a new species collected from Hispaniola is described, for which only undetermined previous records existed, despite several species being known from the neighboring islands of Cuba, Jamaica, and Puerto Rico. *Elpidiumalarconi***sp. nov.** belongs to the group with a basal expansion on the hemipenis distal lobe, which contains six other species (*E.chacoense*, *E.cordiforme*, *E.higutiae*, *E.maricaoense*, *E.merendonense* and *E.picinguabaense*) and can be distinguished from them by the different shape of the basal expansion (appearing long and digitiform) and by a thin, pointed and L-shaped lower ramus. An updated key is constructed to allow identification of the 20 species of *Elpidium* described to date, based on shell morphology and soft parts anatomy. The first sequence of the molecular marker 18S for a described species of *Elpidium* is also presented, and shows its close affinities to members of the genera *Gomphodella*, *Metacypris*, and *Cytheridella*, all in the same clade, separated from the branch where *Limnocythere* is positioned in the phylogenetic tree. These molecular results, together with strong morphological differences, support the promotion of the subfamily Timiriaseviinae to the family rank, independent from the Limnocytheridae, to which it formerly belonged.

## ﻿Introduction

The Ostracoda are a diverse group of crustaceans present in a wide variety of habitats, from deep oceans to mountain springs (Smith and Horne 2002; Mesquita-Joanes et al. 2012). Planktonic ostracods are diverse in marine environments (Angel 1993) but most ostracod species, either marine or nonmarine, are mainly benthic, hypogean or nekto-benthic. However, there are entire groups of species with a very specialized way of life; the Entocytheridae is a speciose family of ostracods exclusively living as symbionts of other crustaceans (Hart and Hart 1974; Mestre et al. 2014); the Terrestricytheridae have the ability to live in humid soils, devoid of a permanent layer of free water (Horne et al. 2004); and members of the genus *Elpidium* are known to dwell in phytotelmata, i.e., very small water bodies accumulating in between plant structures (Jocque et al. 2013).

The most common environments among phytotelmata are tree holes, bromeliads and pitcher plants, and all of them can host relatively simple communities of organisms in tightly organized food webs (Maguire 1971; Kitching 2000). Among the metazoans inhabiting phytotelmata, mosquitoes are possibly one of the most common and more intensely studied groups (Frank 1983). Crustaceans, although less studied than insects, can also be diverse and common, including Copepoda, Decapoda, Ostracoda and Anomopoda (Jocque et al. 2013). Except *Elpidium* ostracods, however, most other species of crustaceans are usually not exclusive from phytotelmata, but rather live in a wide variety of aquatic habitats. Among ostracods living in plant containers, we can also find species that live in other habitats, such as those belonging to the Candonidae, Cyprididae or Darwinulidae (Jocque et al. 2013), but the cytheroid genus *Elpidium* stands out as almost exclusively living in bromeliad phytotelmata (but see Acosta-Mercado et al. 2012), and whose species usually have restricted geographic distributions.

The genus *Elpidium* was established by Müller (1880) to accommodate globular ostracods with a flat ventral surface, which he found very frequently in Brazilian bromeliads: “Ella ali vive em abundancia e quasi que não ha Bromelia sem a sua colonia de Cytherideos; é provavel que, com as Bromelias, ella se estenda por todo o Brazil” (Müller 1881: 27) [“It lives there in abundance, and practically there is no Bromelia without its own colony of cytheroids; probably, as happens with bromeliads, it must be spread all over Brazil”]. No further species of *Elpidium* were described until the middle of the next century (Tressler 1941, 1956). However, during the past few decades, there has been a renewed interest in the genus, and at present we know of 19 described species of *Elpidium* (Pereira et al. 2023; Díaz et al. 2024), distributed in Brazil, Argentina, Honduras, Guatemala, US, Jamaica, Puerto Rico and Cuba, plus some undetermined species from Dominican Republic, Mexico and Costa Rica (Picado 1913; Tressler 1956; Pinto and Purper 1970; Danielopol 1975; Colin and Danielopol 1980; Acosta-Mercado et al. 2012; Pérez et al. 2012; Pinto and Jocqué 2013; Danielopol et al. 2014; Pereira et al. 2019, 2022; Mercado-Salas et al. 2021). The last published key for the identification of *Elpidium* species appeared eleven years ago (Pinto and Jocqué 2013), when only seven species were known to science. With the present survey, we describe for the first time a species of *Elpidium* for the island of Hispaniola and provide an updated identification key including all species described hitherto. In addition, we discuss the biogeography of the genus, and use molecular methods, for the first time in a described species of *Elpidium*, together with morphological data, to evaluate its phylogenetic relationships with other ostracod taxa, supporting the validity of the Timiriaseviidae as a distinct family, separated from Limnocytheridae s.s., as suggested by previous authors (Tanaka et al. 2021).

## ﻿Materials and methods

### ﻿Study area and sampling and laboratory methods

Samples were collected from two localities in the municipality of Jarabacoa (Dominican Republic), in the island of Hispaniola. Jarabacoa is located in La Vega province, in a valley of the Cordillera Central (central ranges) with a mean altitude of 530 m a.s.l. The area is characterized by a siliceous substrate, and wet tropical climate, with an average annual temperature of 20 °C and 1723 mm of mean annual precipitation (Climate-Data.org 2023). The Cordillera Central is included in a single biogeographical area, the Central‐Eastern district, which has one of the highest richness of plant genera and endemic species of Hispaniola (Cano-Ortiz et al. 2017).

Ostracod samples were collected in the frame of a wider survey and various projects on the aquatic invertebrate biodiversity of Hispaniola, which sampled varied habitats, focusing particularly on potential predators of mosquito larvae (Rodríguez Sosa et al. 2019; Olmo et al. 2024). Invertebrate samples were collected by suction from the water stored in between the base of bromeliad leaves, using either a plastic Pasteur pipette, or a 60 mL syringe coupled to a 40 cm flexible hose following Júnior et al. (2017). Most of the Bromeliaceae plants were located in private gardens or nearby, and were tentatively determined as belonging to the genus *Neoregelia*. In the laboratory, the samples were filtered through a 350 μm mesh size filter and fixed in 70% ethanol.

The dissection of ostracod specimens for optical microscopy inspection was done following the protocol described in Namiotko et al. (2011). Soft parts were embedded in HydroMatrix^®^ for the preparation of permanent slides. Shells were stored dry in micropaleontological slides. Drawings were done using a camera lucida on a Leica microscope. Some pictures were taken using a Nikon^®^ Eclipse E800 epifluorescence microscope, either with white light or with UV light (340–380 nm) plus a blue filter (435–485 nm). Some individuals were critical-point dried *in toto* or without the valves. These individuals, plus separated valves of other individuals, were coated with a thin layer of Au-Pd for SEM observation in a Hitachi S-4800 or a SCIOS-2 at the University of València.

### ﻿Taxonomy, chaetotaxy, descriptions, and abbreviations

In this work we follow Mesquita-Joanes et al. (2024) in accepting the suggestion of Tanaka et al. (2021) to raise the subfamily Timiriaseviinae to the family rank, and provide a diagnosis of the family. This diagnosis is established after the differences indicated by Martens (1995) and Danielopol et al. (2018) between Timiriaseviinae and Limnocytherinae. However, most ostracodologists have traditionally considered the Timiriaseviinae as a subfamily within the Limnocytheridae, ever since the review by Colin and Danielopol (1978) (e.g., Savatenalinton et al. 2008; Karanovic and Humphreys 2014; Danielopol et al. 2018; Meisch et al. 2019).

The selection of critical characters to build the identification key was based on those used by Pinto and Jocqué (2013), plus those stressed by Danielopol et al. (2014), supported in some cases by some of the characters included in the phylogenetic tree of Pereira et al. (2022).

Abbreviations used in the text and figures include the following:

**Cp** carapace;

**CL** carapace length;

**H** height of valves;

**L** length of valves;

**LV** left valve;

**RV** right valve;

**W** width of shell;

**A1** antennula;

**A2** antenna;

**Md** mandibula;

**Md-palp** mandibular palp;

**Mx** maxillula;

**T1** first thoracopod;

**T2** second thoracopod;

**T3** third thoracopod;

**CR** caudal ramus;

**Hp** hemipenis;

**DL** distal lobe;

**CoP** copulatory process;

**LR** lower ramus.

Chaetotaxy nomenclature follows mainly Broodbakker and Danielopol (1982), Martens (1987), Meisch (2000), and Pereira et al. (2023). We follow mostly Sames (2011a, 2011b) and Danielopol et al. (2014) for carapace traits terminology. However, terms used for the description of the general shape in dorsal or ventral view of the carapace follow those commonly used for leaves summarized by Hickey (1973), by applying those terms for the tip of leaves to the shape of the anterior part of the carapace, and those for the base of leaves to the shape of the posterior part of the carapace. Note that these terms differ in some cases from those used in the ostracod literature, and in *Elpidium* descriptions in particular, but are more widely used in general in Biology for morphological descriptions.

### ﻿Molecular methods

Ethanol-fixed ostracods were individually transferred to 1.5 mL microtubes using a thin brush. Single specimens from the type locality (e.g., P459=MUVHNZY0040) were digested at 55 °C overnight using 180 µL T1 buffer and 20 µL proteinase K, and DNA was extracted with the Nucleospin DNA extraction kit (Macherey-Nagel™) following the manufacturer’s instructions. The large ribosomal subunit (18S) gene region was amplified using primers 18S_5F 5’-GCG AAA GCA TTT GCC AAG AA-3’ and 18S_9R 5’-GAT CCT TCC GCA GGT TCA CCT AC-3’ (Carranza et al. 1996). Amplifications were carried out using ~ 10 ng of genomic DNA in a reaction containing 1 U of Taq polymerase (Amersham), 1× buffer (Amersham), 0.2 mM of each primer and 0.12 mM dNTPs. The polymerase chain reaction (PCR) thermal profile included an initial denaturation step at 94 °C for 4 min, followed by 30 cycles of 94 °C for 30 s, 50 °C for 30 s, 72 °C for 30 s, and a final extension at 72 °C for 20 min. Sequences were obtained using the Big-Dye Ready Reaction kit v. 3.1 (Applied Biosystems) on an ABI Prism 3770 automated sequencer at the MACROGEN sequencing facilities. Chromatograms for each DNA sequence were checked with BioEdit v. 7.2.5 (Hall 1999) and sequence alignment was conducted with Muscle v. 3.6 (Edgar 2004). Model selection was carried out for the sequence alignment using the Bayesian Information Criterion (BIC) as implemented in ModelTest-NG v. 0.1.7 (Darriba et al. 2020). Maximum likelihood phylogenetic reconstruction was then completed with the corresponding DNA substitution model with ultrafast bootstrap (1000 replicates) as implemented in IQ-TREE v. 2.0 (Minh et al. 2020).

#### ﻿Repository

The holotype, allotype, and paratypes with codes MUVHNZY0021-0042 are deposited in the Natural History Museum of the University of Valencia (**MUVHN**, Burjassot, Spain).

## ﻿Results

### ﻿Taxonomic account

**Class Ostracoda Latreille, 1802**

**Subclass Podocopa G.O. Sars, 1866**

**Order Podocopida G.O. Sars, 1866**

**Suborder Cytherocopina Baird, 1850**

**Superfamily Cytheroidea Baird, 1850**

#### ﻿Family Timiriaseviidae

Taxon classificationAnimaliaPodocopidaLimnocytheridae

Mandelstam, 1960

5DCB932B-3EFF-5F13-AC94-FD7590E072EE

##### Diagnosis.

[Based on the list of characteristic traits of the subfamily Timiriaseviinae by Martens (1995) and Danielopol et al. (2018) and on the types of hinge and sieve pores respectively by Danielopol et al. (2014) and Danielopol et al. (2018)]. Cytheroid Ostracoda with globular shells, particularly in the females, which are larger than males and have a brood pouch (i.e., shell sexual dimorphism apparent). Hinge lophodont, adont, or protodont (Danielopol et al. 2014). Sieve pores absent or type B if present (Danielopol et al. 2018). Terminal segment of the antennula usually short, not longer than the previous segment. Fused part of the antennula Ya aesthetasc with adjacent seta short or not distinguishable, less than one third the length of the aesthetasc. Ventral seta on the second antennular segment situated in a medial or proximal position, not in the distal margin, or absent. Maxillular palp not subdivided in two segments, and with a reduced number of setae. Distal lobe of hemipenis moveable, not fused to the rest of the hemipenis.

#### ﻿Genus Elpidium

Taxon classificationAnimaliaPodocopidaLimnocytheridae

F. Müller, 1880

ACB836A6-6A55-5176-87D4-261BB1F138E7

##### Type species

(by original designation): *Elpidiumbromeliarum* F. Müller, 1880.

##### Type locality.

Itajaí, Santa Catarina state, Brazil.

##### Other species included.

*E.alarconi* sp. nov.; *E.chacoense*Díaz et al., 2024; *E.cordiforme*Pereira et al., 2023; *E.eriocaularum*Pereira et al., 2023; *E.heberti*Pereira et al., 2019; *E.higutiae*Pereira et al., 2023; *E.inaequivalve* Danielopol, 1981; *E.laesslei* (Tressler, 1956); *E.litoreum*Pereira et al., 2022; *E.littlei*Pereira et al., 2019; *E.maricaoense* (Tressler, 1941); *E.martensi*Danielopol et al., 2014; *E.merendonense* Pinto & Jocqué, 2013; *E.oxumae*Pereira et al., 2023; *E.picinguabaense*Pereira et al., 2023; *E.pintoi* Danielopol, 1981; *E.purium*Pereira et al., 2023; *E.purperae* Danielopol, 1981; *E.wolfi*Pereira et al., 2019.

##### Diagnosis.

[Modified after Danielopol et al. (2014) and Pereira et al. (2022, 2023)]. Timiriaseviidae of intermediate size (0.6–1.1 mm) with sexually dimorphic carapace, broad and ventrally flat. Females relatively wider than males, due to the presence of a brood pouch, and usually also larger. Valves symmetric or asymmetric in dorsal view, carapace surface of most species with subtle ornamentation of minute and shallow pits (except *E.laesslei*, which is strongly ornamented). At the mouth part, a funnel structure is internally built in the carapace between both valves. Four apparent adductor muscle scars arranged subvertically (at ~ 15–30° oblique from the vertical axis towards the anterior part from top to bottom). Hinge protodont, with a bar on the smaller valve, which may have prototeeth anteriorly and posteriorly, and a groove in the larger one. A1 apparently six-segmented in most species: with five clearly separated segments, but in most species the fourth segment appears as partially subdivided (4a + 4b). A1 with a dorsal apical expansion in the first segment. A2 sexually dimorphic; three terminal claws in the last segment, one of which is pectinated only in males. Last segment of A2 distally with a small hyaline formation. Mx with two spatulate claws and three normal setae in each of the second and third endites. Hp strongly sclerotized, CR reduced to a pair of setae. Distal lobe very apparent and varied in shape, usually subtriangular or subrectangular, but in some species with a small (pointed or digitiform) expansion in its internal border, always with a basal seta. CoP curved (hook-like, curled, U-shaped or L-shaped), with a tip either subdivided or not in ejaculatory glans and duct. Lower ramus varied in shape. Upper ramus absent. Female abdomen rounded, with a dorsal spine-like seta, sclerotized genital lobes, and three setae on each CR lobe.

#### ﻿ Elpidium alarconi sp. nov.

Taxon classificationAnimaliaPodocopidaLimnocytheridae

BA7FC90E-2C60-541C-A56E-BAC1FE2F7BEF

https://zoobank.org/D973DE83-200E-4BEF-AE4D-05174A5FD155

**Figure 1.**
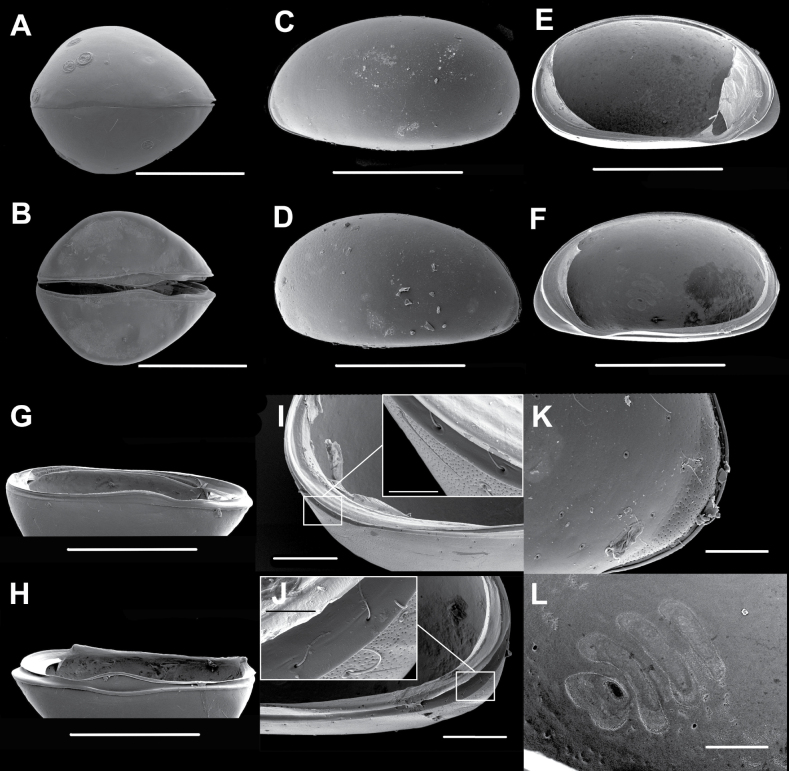
*Elpidiumalarconi* sp. nov. ♂ **A**Cp dorsal view (MUVHNZY0026) **B**Cp ventral view (MUVHNZY0025) **C**LV, external view (MUVHNZY0035) **D**RV external view (MUVHNZY0035) **E**LV internal view (MUVHNZY0036) **F**RV internal view (MUVHNZY0036) **G**LV subventral view (MUVHNZY0036) **H**RV subventral view (MUVHNZY0036) **I**LV internal view (MUVHNZY0036), detail of posterior part, and zoom on lateral row of pores (inset) **J**RV internal view (MUVHNZY0036), detail of posterior part, and zoom on lateral row of pores (inset) **K**Cp detail anterior part, right external view (MUVHNZY0035) **L** Detail adductor muscle scars, RV internal view (MUVHNZY0036). Scale bars: 400 μm (**A–H**); 100 μm (**I, J** general); 20 μm (**I, J** inset); 50 μm (**K, L**).

**Figure 2.**
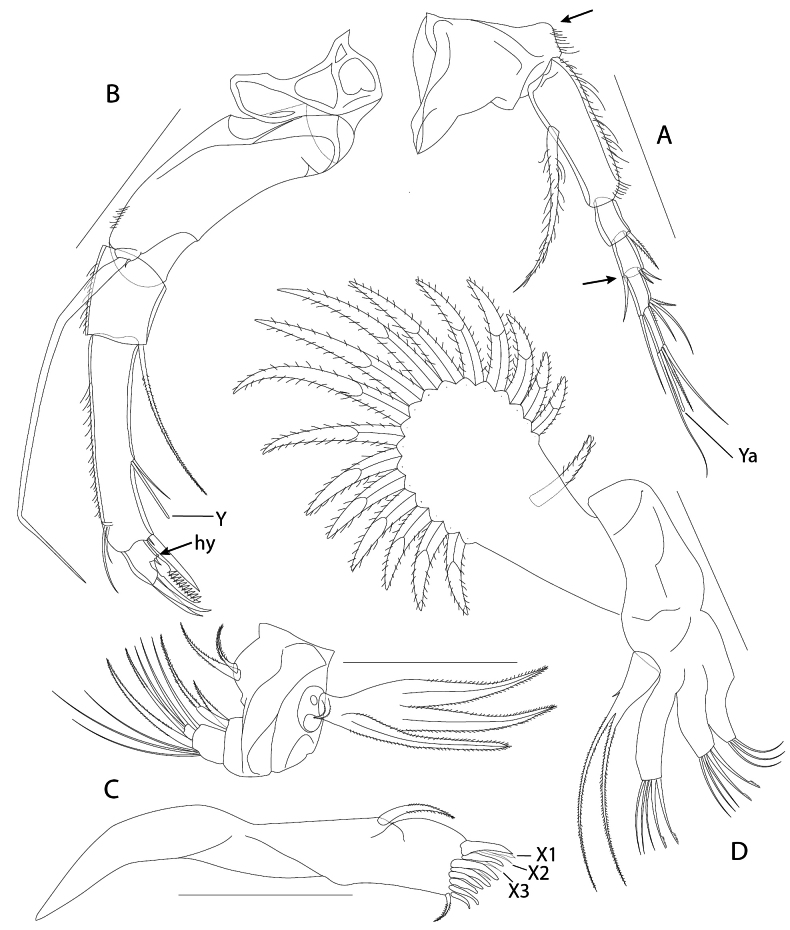
*Elpidiumalarconi* sp. nov. ♂ (MUVHNZY0021) **A**A1, top arrow points to subtriangular expansion on first segment; bottom arrow points to the partial separation between segments 4a and 4b **B**A2, hy: hyaline formation **C**Md palp (top) and coxa (bottom) **D**Mx. Scale bars: 100 μm.

**Figure 3.**
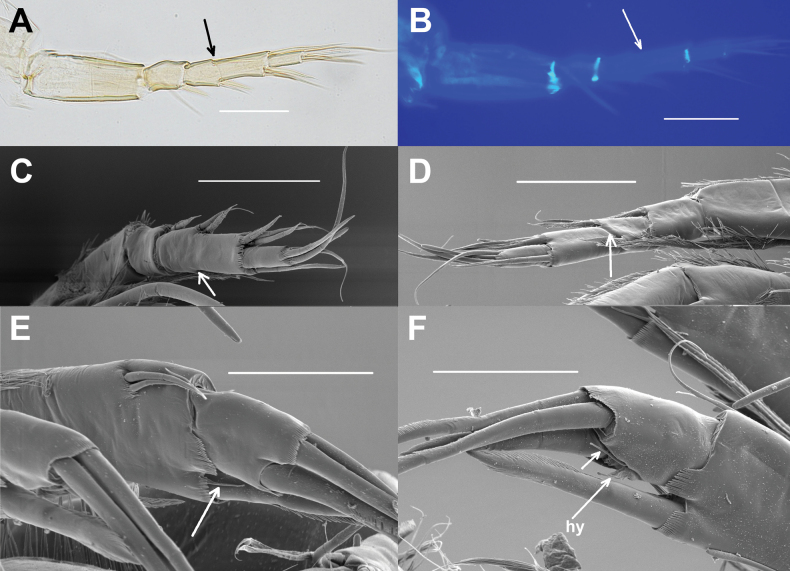
*Elpidiumalarconi* sp. nov. details of A1 and A2**A**A1, arrow points to the partial separation between segments 4a and 4b. Picture in white transmitted light. ♂ (MUVHNZY0035) **B**A1, arrow points to the partial separation between fourth and fifth segment (4a and 4b). Picture using UV light and blue filter in a fluorescence microscope. ♂ (MUVHNZY0035) **C** SEM image of right A1, external view; arrow points to the lack of separation between fourth and fifth segment (4a and 4b). ♂ (MUVHNZY0039) **D** SEM image of right A1, internal view; arrow points to the separation between fourth and fifth segment (4a and 4b) (Same individual as in C:MUVHNZY0039) **E** SEM image of left A2, internal view; arrow points to a ventroapical minute seta on the penultimate segment. ♀(MUVHNZY0038) **F** SEM image of left A2, external view; one arrow points to a ventroapical minute seta on the last segment, and another to the (crumpled) hyaline formation (hy). ♀ (MUVHNZY0038). Scale bars: 50 μm (**A–D**); 30 μm (**E, F**)

**Figure 4.**
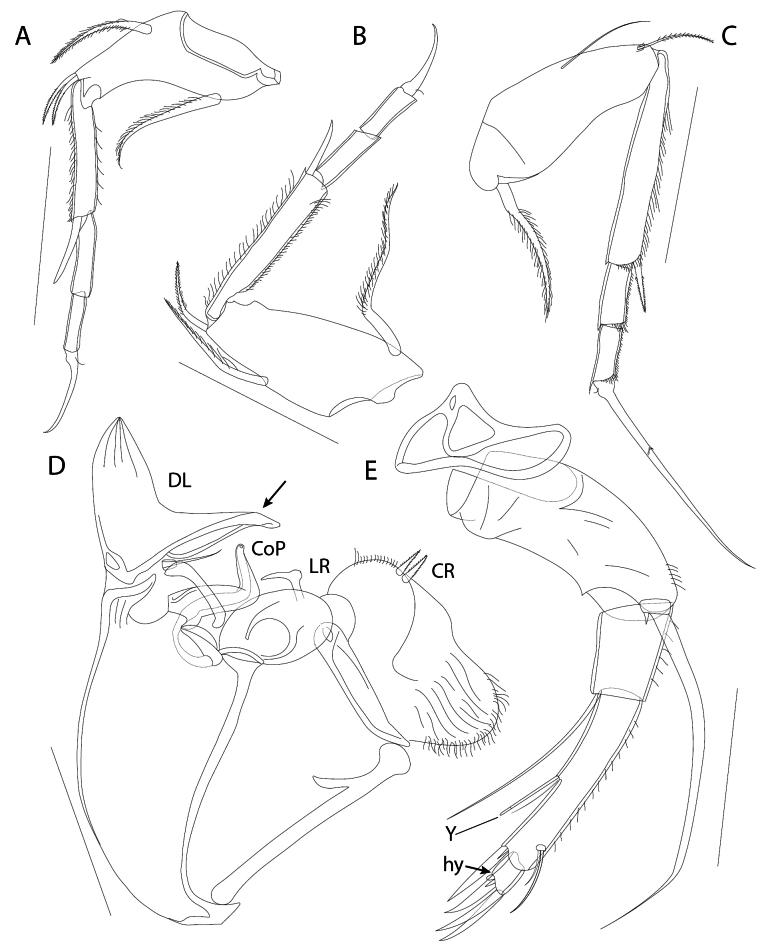
*Elpidiumalarconi* sp. nov. ♂ (**A–D**) (MUVHNZY0021) and ♀ (**E**) (MUVHNZY0037) **A**T1**B**T2**C**T3**D**Hp**E**A2; hy: hyaline formation. Scale bars: 100 μm.

**Figure 5.**
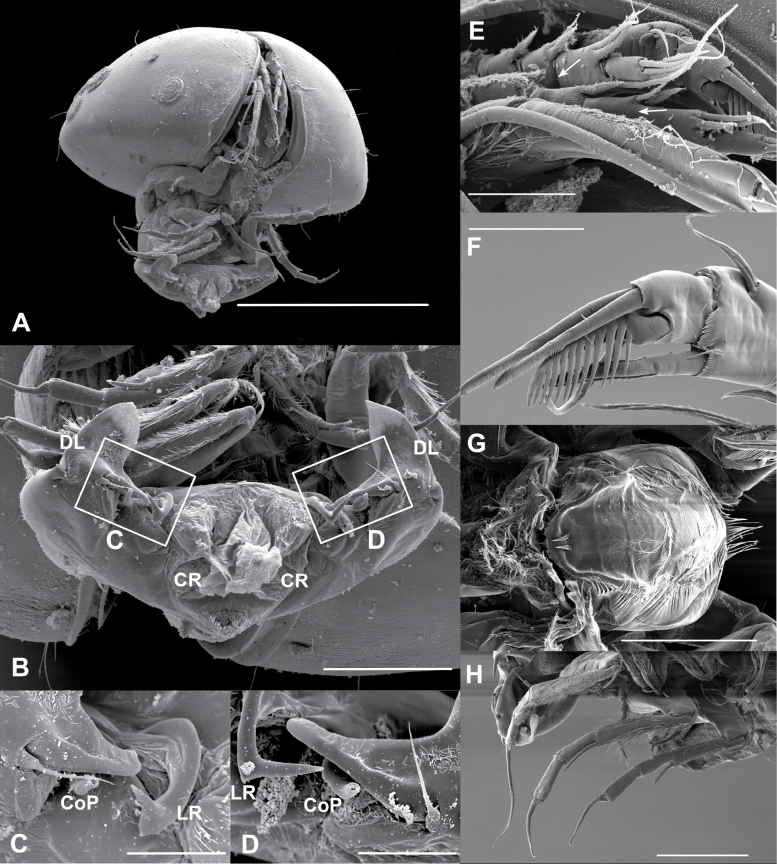
*Elpidiumalarconi* sp. nov. ♂ **A–E** MUVHNZY0027 **F–H** MUVHNZY0039 **A** complete frontal view of individual with extended penis **B** detail of penis **C** detail of digital expansion of DL, CoP and LR (right hemipenis) **D** detail of digital expansion of DL, CoP and LR (left hemipenis) **E** detail of A1 showing separation between segments 4a and 4b in internal part of left A1 (leftmost arrow) and the lack of separation between them in the external part of right A1 (right arrow) **F** detail of right A2 distal part (internal view) **G** labrum, ventral view **H** left T1-T3. Scale bars: 400 μm (**A**); 100 μm (**B, G, H**); 30 μm (**C, D, F**); 40 μm (**E**).

**Figure 6.**
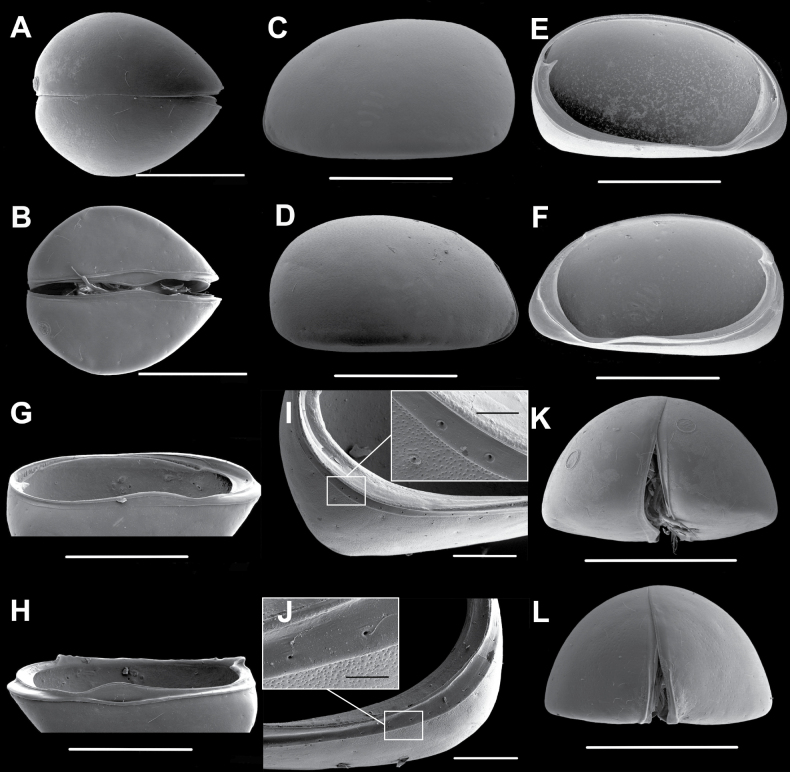
*Elpidiumalarconi* sp. nov. ♀ **A**Cp dorsal view (MUVHNZY0028) **B**Cp ventral view (MUVHNZY0029) **C**LV, external view (MUVHNZY0037) **D**RV external view (MUVHNZY0037) **E**LV internal view (MUVHNZY0037) **F**RV internal view (MUVHNZY0037) **G**LV subventral view (MUVHNZY0037) **H**RV subventral view (MUVHNZY0037) **I**LV internal view (MUVHNZY0037), detail of posterior part, and zoom on lateral row of pores (inset) **J**RV internal view (MUVHNZY0037), detail of posterior part, and zoom on lateral row of pores (inset) **K**Cp posterior view (MUVHNZY0031) **L**Cp anterior view (MUVHNZY0032). Scale bars: 400 μm (**A–H, K, L**); 100 μm (**I, J** general); 20 μm (**I, J** inset).

**Figure 7.**
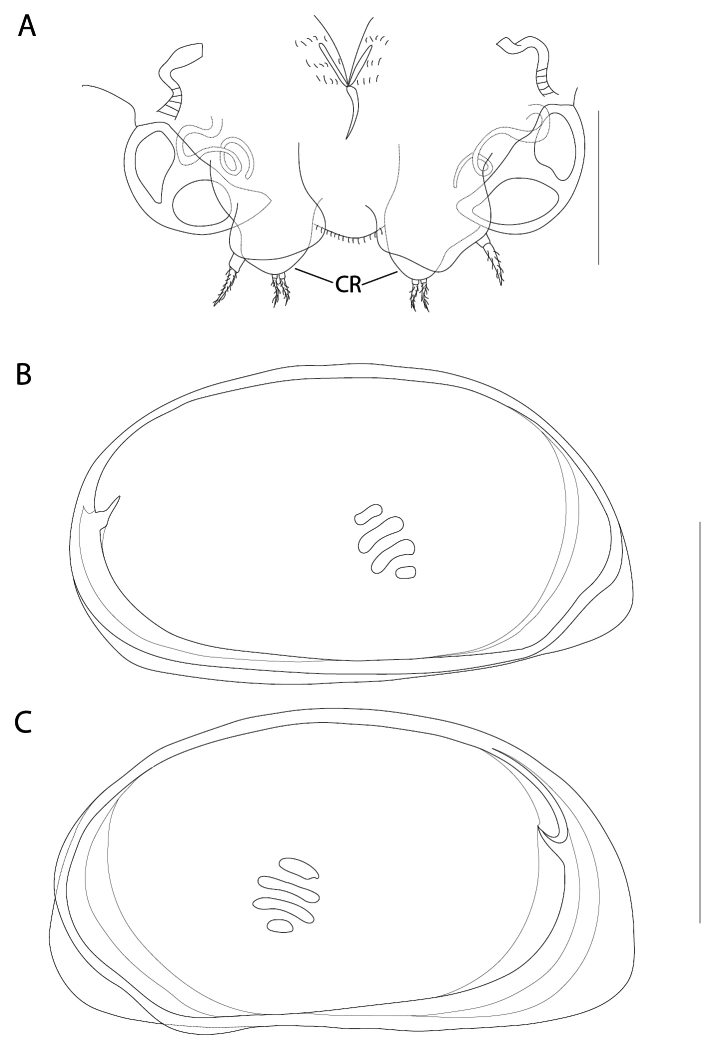
*Elpidiumalarconi* sp. nov. ♀ **A** Posterior part of abdomen (MUVHNZY0022) **B**LV internal view (MUVHNZY0033) **C**RV internal view (MUVHNZY0033). Scale bars: 100 μm (**A**); 500 μm (**B, C**).

Figs 1 , 2 , 3 , 4 , 5 , 6 , 7

##### Type locality.

Rancho Baiguate (La Joya Sector, Jarabacoa, República Dominicana) 19°6'49"N, 70°37'8"W, 530 m a.s.l., sampled on 7/2/2019 and 12/4/2021 by J. Rueda and P. Alarcón. Tank bromeliads growing at the base of several tree trunks in a secondary natural forest, with a wide cover, and presence of domestic animals (horses, dogs) in the vicinity, near the Baiguate River.

##### Type material.

***Holotype*** • 1 adult ♂; soft parts dissected and stored on a permanent microscopic slide, valves dry in a micropaleontological slide (MUVHNZY0021). ***Allotype*** • 1 adult ♀; soft parts dissected and stored on a permanent microscopic slide, valves dry in a micropaleontological slide (MUVHNZY0022). ***Paratypes*** • 10 adult ♂♂ and 17 adult ♀♀. Six of the males (MUVHNZY0023 - MUVHNZY0026, MUVHNZY0035, MUVHNZY0036) dissected and stored as the holotype, valves coated and used for SEM; one male (MUVHNZY0027) used *in toto* for SEM, after applying critical-point drying (CPD), and stored in a micropaleontological slide; another male (MUVHNZY0039) with valves untreated and bodies (CPD and coated) in a micropaleontological slide. Seven females (MUVHNZY0028-0033, MUVHNZY0037) dissected and stored as the holotype, valves coated and used for SEM; another female (MUVHNZY0038) with valves untreated and bodies (CPD and coated) in a micropaleontological slide. Two adult males and six females stored *in toto* in ethanol 96% (MUVHNZY0034). Soft body remains of three adult females used for DNA extraction stored in ethanol (codes MUVHNZY0040-0042).

##### Diagnosis.

*Elpidium* species of intermediate size (~ 700–800 μm), with a dark-colored carapace. Females slightly longer and wider than males, and with a truncate posterior margin in dorsal view; males with a barely obtuse posterior margin. Valves (quasi-)symmetric in dorsal view. Surface of valves covered with minute and shallow pits. LV embracing RV along all free margins. Hinge protodont, with a strongly built bar in the RV, including one (proto-)tooth at each extreme of the bar. LV with a hinge groove. A1 apparently six-segmented (i.e., segments 4a and 4b partially separated). DL of male Hp with a long digital expansion, CoP L-shaped, with tip not subdivided, and LR very slender (thinner than CoP), L-shaped and with a pointed tip.

##### Description.

**Male.** Adult shell large (L > 0.7 mm), according to size groups established for limnocytherids s.l. by Gidó et al. (2007), but of intermediate size compared to other *Elpidium* species. Cp subovate in dorsal and ventral view (Fig. 1A, B). Maximum width slightly displaced to posterior part, at ~ 45% of total length. Cp in dorsal view: anteriorly pointed, barely acute; posteriorly bluntly pointed, obtuse, with more rounded outline than anterior margin. Valves almost symmetrical in dorsal view; LV slightly longer and embracing RV along all free margins (Fig. 1B). Valves elongate in lateral view (Fig. 1C, D), posterior margin broadly rounded, anterior margin infracurvate, i.e., narrowly rounded towards anteroventral region. Maximum length at ~ 33% of maximum height. Ventral margin slightly convex in lateral view, flat in ventral (Fig. 1B) and frontal (Fig. 5A) views. Surface of valves smoothly punctate, overall covered with minute foveolae and sparsely with normal (type-A2) pores, many of which hold a sensory seta (Fig. 1A, C, D, I–K). These foveolae more conspicuous, denser, and deeper near anterior margin of valves, in a narrow beak-like zone (Fig. 1K). This zone partially corresponds internally to the area of the inner lamella between outer margin and selvage (Fig. 1E, F). Calcified inner lamella wider anteriorly (~ 12% of valve L) than posteriorly (6% of valve L). Selvage strongly built in the RV (Fig. 1F, H), anteriorly positioned approximately half way between anterior margin and inner margin of calcified inner lamella. Hinge protodont, sensu Danielopol et al. (2014). RV dorsally with a hinge bar (Fig. 1H), showing anterior and posterior prototeeth. LV with a hinge groove (Fig. 1G), anteriorly with enlarged socket. Both valves antero-ventrally with selvage protruding towards external margin, building the typical funnel-like structure of *Elpidium* ostracods at mouth position. Four large adductor muscle scars (Fig. 1F, L) aligned in a slightly oblique row (leaning 30° from vertical axis towards anterior part, from top to bottom), located just in front of central area of valves. Three of these scars elongate, bottom one subovate. Another smaller, rounded scar situated in front of top one of the four central muscle scars. Both valves postero-ventrally with a row of submarginal (type-A2) pores and setae located in the peripheral part of the marginal infold (Fig. 1I, J). Carapace colored dark brown.

A1 (Figs 2A, 3A–D). Apparently six-segmented, i.e., with clear separation between segments 4a and 4b under standard microscope, but this separation weaker than other segments (Fig. 3A). Separation not observed under UV-light in a fluorescence microscope, compared to other segmentation (Fig. 3B). This separation observed only in the internal part of fourth segment under SEM, but not in the external part (Figs 3C, D, 5E). First segment trapezoidal, strongly built, dorsally with a subapical subtriangular expansion, partially covered with pseudochaetae. Second segment elongate, more than thrice longer than wide, dorsally covered with pseudochaetae along its margin, ventrally with a long plumose seta, attached slightly behind middle of segment, and reaching mid-length of fourth segment. Third segment rectangular, with a seta at its dorso-apical margin, this seta slightly longer than next segment. Segment 4a rectangular, ~ 2× longer than wide, dorsally with two small apical setae (not attaining the middle of next segment) and ventrally one seta as long as next segment. Fifth segment (segment 4b) dorsally with three apical setae of varied length; longest one attaining one third of Ya aesthetasc, second longer one as long as last segment, smallest one ~ 1/2 the length of last segment. Ventrally with a long apical seta, surpassing the middle of Ya aesthetasc. Last (sixth) segment with three apical setae and Ya aesthetasc. One seta as long as Ya, another slightly longer than last two segments, another one slightly longer than last three segments.

A2 (Fig. 2B). Protopod two-segmented. First segment short and ring-shaped, second segment elongate and smoothly curved, > 2.5× longer than wide. Exopod with a very small seta and a spinneret seta, not surpassing tip of claws. First segment of endopod subquadrate, ventrally with an apical long seta, ~ 2/3 of the length of next segment. Second endopodal segment elongate, ~ 5× longer than wide. Ventrally with one small seta and Y aesthetasc, situated slightly in front of mid-length of segment. This small seta slightly shorter than aesthetasc. Another large and thick seta attached to ventro-apical margin, together with a minute seta (Fig. 5F, as in the female: Fig. 3E). Dorsally with two subapical short setae, one ~ 1/2 the length of the other. Last segment subquadrate, with three claws, shortest and ventral one pectinated with a row of strong teeth (Fig. 5F). A very small hyaline formation located ventro-apically, at the base of pectinated claw, but together with a minute seta (as in the female: Fig. 3F).

Md (Fig. 2C). Coxa slender, with curved posterior half and straight anterior one. Distally with eight teeth, progressively smaller from anterior (dorsal) to posterior (ventral) ones, most of them bicuspidate and/or with adjacent interdental spines and setae (X-setae). Dorsally with large serrate seta, not reaching the base of dorsal teeth. Ventrally with one small plumose seta, slightly longer than ventralmost small tooth. Md-palp four-segmented and curved. First segment (basis) with two ventral plumose setae, one ~ 2/3 the length of the other. Dorsally with exopod (respiratory plate) with three broad rays and a small reflected ray. Second segment (first endopodal segment) with two ventro-apical plumose setae, one of them half the length of the other. Third segment subquadrate, ventrally holding an apical long smooth seta, dorsally with three long apical smooth and thin setae, together with a thicker plumose seta, all of similar length. Last segment small and subquadrate, with three terminal thin setae of similar length, one of these claw-like, the other two smooth.

Mx (Fig. 2D). Elongate, subrectangular protopod. Exopod (respiratory plate) with 16 distal unequal rays and a proximal reflexed ray. Endopod with three endites and a palp. First endite with three subequal setae. Second and third endites each with two spoon-shaped (spatulate), claw-like setae, and three smooth, thin setae. Palp unsegmented, distally with two long plumose setae, longer than tip of endite setae, plus a minute subapical dorsal seta.

T1 (Figs 4A, 5H). Four-segmented. First segment the longest. Ventrally with a large seta, situated well behind mid-length of segment. Dorsally with proximal long seta, slightly surpassing distal margin of segment. Dorso-apically with two subequal knee-setae. Second segment elongate, 6× longer than wide, ventrally with strong apical seta, as long as next segment. Third segment without setae. Fourth segment with apical claw bearing a minute seta at its swollen base, and as long as third segment.

T2 (Figs 4B, 5H). Larger than T1 and four-segmented. First segment strong, bearing ventrally a subproximal long setae, attaining distal edge of segment. Dorsally with one medial long seta, surpassing distal margin of segment, and an apical knee-seta, ~ 1/2 the length of previous seta. Second segment slender and long, ventrally with apical strong seta, almost as long as next segment. Third segment without setae. Fourth segment similar to previous one but slightly shorter and with an apical claw. This claw as long as third segment, and with a proximal minute seta.

T3 (Figs 4C, 5H). Larger than T2 and four-segmented. First segment ventrally with a proximal large seta, 2/3 as long as segment. Dorsally with a thin medial seta, attaining distal edge of segment, and a small distal knee-seta, ~ 1/2 the length of previous seta. Second segment long, > 8× longer than wide, and with an apical strong seta, ~ 2/3 the length of next segment. Third segment devoid of setae and 3× longer than wide. Last segment similar but slightly smaller than previous one, bearing a very long claw, longer than second segment, and with a minute seta at its base.

Hp (Figs 4D, 5A–D). Large sclerotized and muscular body with DL, distal seta, CoP and LR. DL with a long basal digital expansion. Width of DL, including digital expansion, longer than its length. This expansion flexible at its tip, so that in some slide preparations for optical microscopy, it can be distally folded. Distal seta shorter than digital expansion. DL with lateral margins almost parallel in its mid length, but converging in a subtriangular, pointed shape at its distal part (Figs 4D, 5B). CoP L-shaped, progressively narrowing towards the tip (Fig. 4D), without separation between distal glans and ejaculatory duct (Fig. 5C, D). LR slender, very thin, L-shaped and with a finely pointed tip (Figs 4D, 5C, D). Depending on the position of LR in slide preparations for optical microscopy, L-shape might not be seen clearly in one or both hemipenes. A slight difference between left and right LR shape observed in the development of the L-angle, somehow resembling a piolet with a small adze rather than an L (Fig. 5C, D).

CR with one pair of intermediate-size, plumose setae and numerous pseudochaetae (Figs 4D, 5B).

Labrum (Fig. 5G) large, subquadrate in ventral view. Anteriorly and ventro-laterally with arrays of long pseudochaetae. Posteriorly, near the mouth entrance, with two submarginal pappose setae and a marginal row of short setulae forming an apparently serrated margin.

##### Description.

**Female** (only sexually dimorphic features described) (Figs 6, 7). Cp slightly longer, distinctly wider, and slightly more asymmetric than male, posteriorly not pointed but truncate or even slightly cordate in dorsal and ventral views (Fig. 6A, B). These Cp differences between male and female correspond to species group A, according to Danielopol et al. (2014). In lateral external view (Fig. 6C, D), female valves with a straight ventral margin and a less arched posterior margin than males. In internal view, more developed socket-like hinge structures posteriorly in the inner margin of both valves (Figs 6E–H, 7B, C), and posteroventrally wider distance between outer margin and external outline, due to the wider development of valves in this area (Fig. 6E, F, I, J). Posterior part of female hinge bar also with stronger tooth, coupled to a tooth-like pointed inner margin in RV (Fig. 6F, H), not observed in male valves (Fig. 1H).

A2 (Figs 3E, F, 4E). None of the three claws in distal segment pectinated. Y aesthetasc smaller than in male, i.e., of similar length than adjacent seta.

Abdomen (Fig. 7). Centrally with a spine-like seta in dorsal position. Genital lobes semicircular, with internal trabecula and showing internal tubes. CR with two equal adjacent plumose setae in an apical position plus a separate stronger plumose seta, laterally situated, close to genital lobe.

##### Measurements.

**Male.** L: 739 μm (671–778, *n* = 7); W: 559 μm (524–596, *n* = 5); H: 423 μm (418–430, *n* = 3). **Female.** L: 773 μm (711–836, *n* = 9); W: 645 (556–711, *n* = 5); H: 422 (373–476; *n* = 4).

##### Differential diagnosis.

Other *Elpidium* species with similar Cp, i.e., with LV embracing RV, symmetric in dorsal view, not ornamented and with sexual dimorphism of group A, include *E.bromeliarum*, *E.pintoi*, *E.littlei*, *E.litoreum*, and *E.purium*, but none of these species have a digital expansion at the base of the DL, although *E.littlei* has some subdigitiform, elongate triangular expansion. The species *E.maricaoense* and *E.merendonense* have a similar digital expansion (although smaller than in *E.alarconi* sp. nov.), but their Cp are asymmetrical in dorsal view. The Brazilian species *E.cordiforme* has a similar digital expansion, but its Cp is strongly cordiform in dorsal view, and the CoP and LR of Hp are notably different. Another Brazilian species, *E.picinguabaense* and the Argentinian *E.chacoense* also have a digital expansion in the DL. However, this expansion is shorter than in the new species. In addition, the female Cp of *E.picinguabaense* is not posteriorly truncate, but narrowly rounded, and the LR of the Hp is distinctly larger than in *E.alarconi* sp. nov. The female Cp of *E.chacoense* is not truncate posteriorly in dorsal view, but barely pointed. *Elpidiumhigutiae*, also from Brazil, has a similar Cp shape to *E.alarconi* sp. nov., and it also has a digital expansion on the DL, but this expansion is shorter than in *E.alarconi* sp. nov. and its LR is larger and thicker than in the new species. In fact, the very thin L-like shape of the LR in *E.alarconi* sp. nov. is a unique trait that allows distinction from all other *Elpidium* species.

##### Ecology and distribution.

Besides the type locality of Rancho Baiguate, it has also been found in Pinar Dorado Hotel (19°7'2"N, 70°37'58"W), 549 m a.s.l., sampled on 20 March 2018 by J. Rueda and P. Alarcón. This site is in the same municipality of Jarabacoa, but in the Pinar Dorado Sector. Tank bromeliads (possibly of the genus *Neoregelia*) growing at the base and the trunk of several trees in a relatively anthropized habitat composed of a law garden surrounded by pine trees, with a pool and a bar located nearby. In the type locality, the species was collected from the same type of bromeliads. Paratypes MUVHNZY0035, MUVHNZY0036, and MUVHNZY0037 were collected from this locality; other types were collected in the type locality.

##### Etymology.

The species is named after Dr. Pedro María Alarcón-Elbal, who organized the sampling campaign in República Dominicana, obtained financial support and encouraged the senior author JR to study the invertebrates of the area.

### ﻿Molecular phylogeny

**Figure 8.**
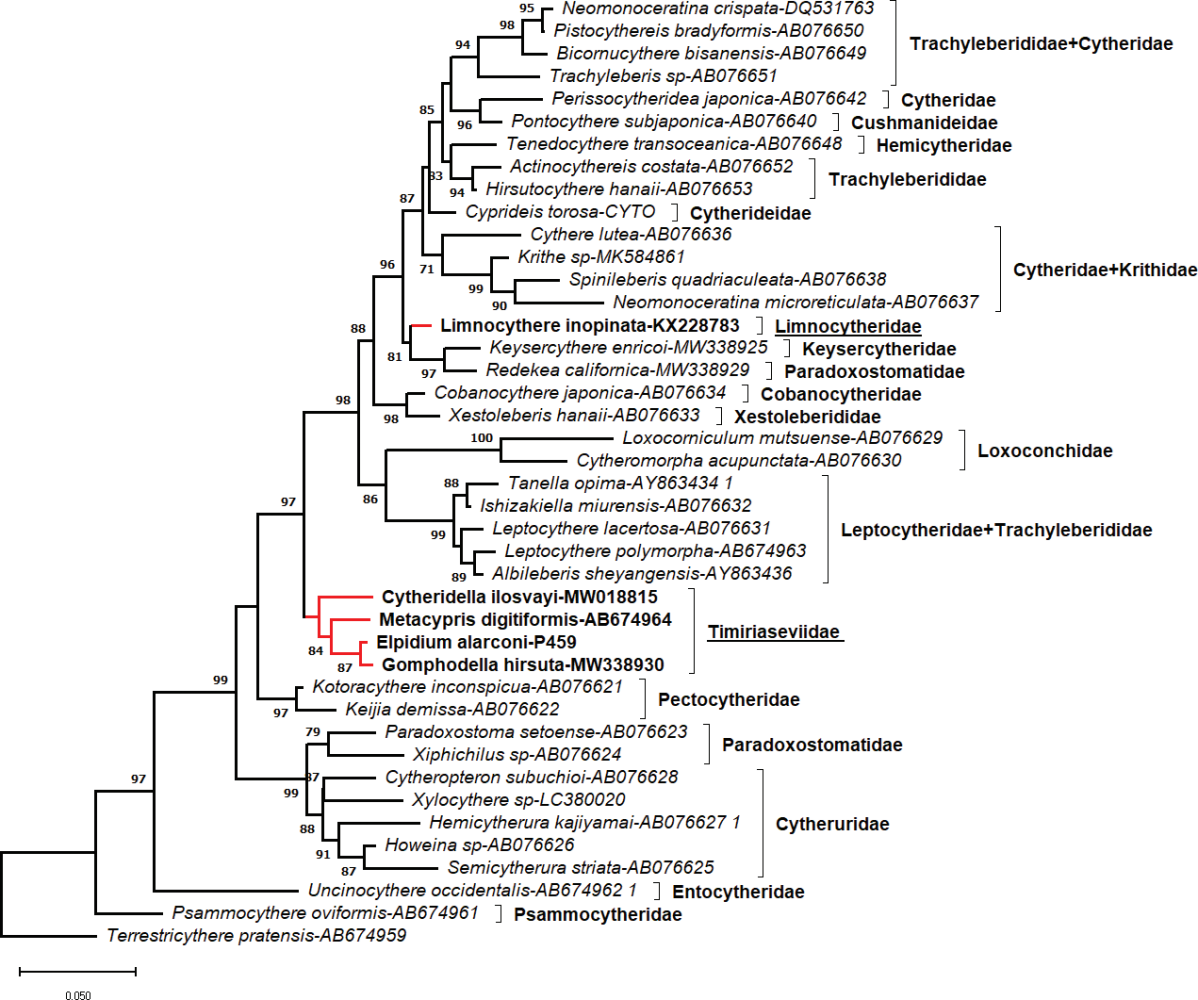
Maximum Likelihood tree inferred using the GTR model on the 18S rDNA alignment data. Bootstrap support values (percentage of trees in which the associated taxa clustered together) larger than 70 are shown next to the branches. The tree is drawn to scale, with branch lengths measured in the number of substitutions per site. Specimen *Elpidium*-P459 stored as paratype with code MUVHNZY0040 in the repository.

We have obtained new 18S rDNA sequences for *Elpidiumalarconi* sp. nov. and *Cyprideistorosa*, with GenBank accession numbers PP648174 and PP648175, respectively. The 18S rDNA sequence alignment had 739 bp in length and followed the GTR substitution model according to BIC model selection. The phylogenetic tree obtained (Fig. 8) placed *Elpidiumalarconi* sp. nov. in the same clade as *Metacypris*, *Cytheridella*, and *Gomphodella*, all of them belonging to the Timiriaseviidae (formerly subfamily Timiriaseviinae). This clade becomes clearly separated from the genus *Limnocythere*, and therefore the Limnocytheridae s.s. Interestingly, the clade formed by the Timiriaseviidae genera, holds a more basal position within the Cytheroidea, splitting earlier than Limnocytheridae, but also than other families, including Xestoleberididae, Loxoconchidae, Cytheridae and Cytherideidae, among others.

### ﻿Identification key to species of *Elpidium*

**Table:** 

1	RV ventrally overlapping LV	**2**
–	LV ventrally overlapping RV	**4**
2	Female CL = 0.7–0.9 mm. Hp: DL lateral margins parallel or convergent from base to mid-length	**3**
–	Female CL ≥ 0.9 mm. Hp: DL lateral margins divergent from base to mid-length	***E.oxumae***
3	Female Cp in dorsal view: greatest width at posterior half of CL. Hp: DL tip blunt, DL lateral margins parallel or slightly convergent from base to mid-length	***E.martensi***
–	Female Cp in dorsal view: greatest width at mid-length of CL. Hp: DL tip acute, DL lateral margins convergent along its whole length	***E.purperae***
4	Cp surface smooth or covered with minute foveolae	**5**
–	Cp surface ornamented	***E.laesslei***
5	Female CL ≥ 0.9 mm	**6**
–	Female CL < 0.9 mm	**8**
6	Female Cp in dorsal view rounded (CL:W ≤ 1.2), symmetrical, with posterior margin truncate or cordate. Hp: DL tip blunt, CoP tip undivided	**7**
–	Female Cp in dorsal view elongate (CL:W > 1.2), asymmetrical, with posterior margin pointed, barely obtuse, almost acute. Hp: DL tip acute, CoP tip divided	***E.heberti***
7	Female Cp in dorsal view: posterior margin truncate, greatest width at mid-length. Hp: DL lateral margins divergent from base to mid-point, DL with a small distal or medial subtriangular expansion	***E.bromeliarum***
–	Female Cp in dorsal view: posterior margin cordate, greatest width at posterior half of Cp. Hp: DL lateral margins convergent at mid-length, DL with a basal digitiform expansion, longer than basal seta	***E.cordiforme***
8	Female CL ≤ 0.7 mm	**9**
–	Female CL 0.7–0.9 mm	**11**
9	Female Cp symmetric in dorsal view	**10**
–	Female Cp asymmetric in dorsal view	***E.inaequivalve***
10	Female Cp in dorsal view: posterior margin truncate. Hp: right DL tip blunt, left DL tip acute, basal digitiform expansion absent	***E.litoreum***
–	Female Cp in dorsal view: posterior margin pointed, barely obtuse. Hp: DL tip acute, basal digitiform expansion present	***E.picinguabaense***
11	CoP tip divided	**12**
–	CoP tip undivided	**13**
12	Female Cp in dorsal view rounded (CL:W ≤ 1.2), symmetrical, posterior margin rounded. Hp: DL tip blunt, DL basal digitiform expansion present and pointed	***E.littlei***
–	Female Cp in dorsal view elongate (CL:W > 1.2), asymmetric, posterior margin pointed, barely obtuse, almost acute. Hp: DL tip acute, DL basal digitiform expansion absent	***E.wolfi***
13	Hp: DL internally with digitiform expansion	**14**
–	Hp: DL without internal expansion or, if present, not digitiform but a short pointed subtriangular expansion	**18**
14	Female Cp in dorsal view symmetrical	**15**
–	Female Cp in dorsal view asymmetrical	**17**
15	Female Cp posterior margin pointed (obtuse) in dorsal view	***E.chacoense***
–	Female Cp posterior margin truncate in dorsal view	**16**
16	Female Cp rounded in dorsal view (CL:W ≤ 1.2), greatest width at mid length. Hp: lower ramus with a thin pointed tip. Digitiform expansion longer than basal seta	***E.alarconi* sp. nov.**
–	Female Cp elongate in dorsal view (CL:W > 1.2), greatest width at posterior half of Cp. Hp: lower ramus with a broad pointed tip. Digitiform expansion shorter than basal seta	***E.higutiae***
17	Female CL:W ≤ 1.2, DL finger shorter than basal seta	***E.maricaoense***
–	Female CL:W > 1.2, DL finger long (as long or longer than basal seta)	***E.merendonense***
18	Hp: DL lateral margin without any expansion	***E.pintoi***
–	Hp: DL lateral margin with a subtriangular expansion	**19**
19	Female CL < 0.8 mm. Hp: DL lateral pointed expansion at mid-length, basal lateral margins divergent	***E.eriocaularum***
–	Female CL > 0.8 mm. Hp: DL lateral pointed expansion at more distal position than mid-length, basal lateral margins almost parallel	***E.purium***

## ﻿Discussion

### ﻿Morphology and similar species

*Elpidiumalarconi* sp. nov. has a shell morphology that does not differ widely from those of other *Elpidium* species with symmetric smooth valves, shell closure with left valve embracing right valve, and truncate posterior margin in dorsal view, such as *E.higutiae*, *E.purium*, *E.litoreum*, *E.pintoi* or even the type species *E.bromeliarum*. Yet, some of these species are either larger, as *E.bromeliarum*, or more elongated (*E.higutiae*, *E.litoreum*). The remaining two species, *E.purium* and *E.pintoi*, are very similar in dorsal view and their carapace sizes overlap with that of *E.alarconi* sp. nov. However, both lack a basal digitiform expansion in the distal lobe of the hemipenis, which is present, and very long, in the new species. This relatively straightforward distinction between species could be established thanks to a previous review of the variability of morphological traits in the genus *Elpidium* by Danielopol et al. (2014). These authors highlighted the importance of valve surface (smooth or ornamented), shell size and closure (left or right valve overlapping the other one), and its outline in dorsal view, including symmetry or asymmetry of valves, shape of posterior margin (pointed, rounded, truncate, invaginated), and length/width relationship. These traits are very useful for morphological characterization of *Elpidium* species, and therefore for identification keys, so we also used them in the new key provided, which now includes 20 described species. However, besides the indication of a pointed shape, we used the more precise term “obtuse” for an angle > 90°, and “acute” for an angle < 90°, and rather than “invaginated”, we used the term “cordate”, following Hickey (1973). We call for a more general use of this terminology, well established in the literature for leaf shape, but which can be also applied to ostracod shape in dorsal or ventral view.

In some cases, carapace morphology alone is not enough to easily distinguish between similar species, and other characters may be needed. Indeed, the most diversified morphological trait in *Elpidium* ostracods is the shape of the hemipenis and, in particular, that of its distal lobe, copulatory process and lower ramus (Danielopol 1975; Danielopol et al. 2014; Pereira et al. 2022). Hemipenis morphology has long been considered an essential character in ostracod phylogeny, allowing species determination in lineages with similar shell structure (Danielopol 1969; Hart and Hart 1974; Bisquert-Ribes et al. 2023), and this seems to be also the case in the genus *Elpidium*. Besides *E.alarconi* sp. nov., there are other species that also have a basal digitiform expansion in the distal lobe of the hemipenis: *E.chacoense*, *E.cordiforme*, *E.picinguabaense*, *E.merendonense*, *E.maricaoense*, and *E.higutiae*. But out of these, this expansion is as long or longer than the basal seta of the distal lobe only in *E.alarconi* sp. nov., *E.cordiforme*, and *E.merendonense*. It is nevertheless distinctly shorter in *E.merendonense* than in the other two species, and this species is furthermore distinguished because of an asymmetric carapace shape in dorsal view, and a lower ramus of the hemipenis with a blunt tip. Despite the similarity of the digital expansion of *E.cordiforme* with that of the new species, its distal lobe has a blunt tip (pointed in the new species). In addition, *E.cordiforme* has a cordate posterior margin in dorsal view (hence its name), whereas *E.alarconi* sp. nov. has a truncate posterior margin, although a slight invagination (i.e., quasi-cordate shape) can be appreciated in some shells. Together with the distal lobe shape, the morphology of the lower ramus is also remarkable in the new species, as it is thinner than in any other member of the genus, and L-shaped, somehow resembling a piolet or a very thin sock with an acuminate tip. The lower rami are also pointed and even almost L-shaped in other species of *Elpidium*, but always thicker at the basal part, as for instance in *E.higutiae*, *E.maricaoense*, *E.oxumae*, or *E.cordiforme*. Taking these hemipenis characters into account, *E.cordiforme* is one of the species closer to *E.alarconi* sp. nov., although the former has a twisted copulatory process, unlike any other species of the genus. Furthermore, the distal lobe of *E.merendonense* and the lower ramus *of E.maricaoense* are the most similar hemipenis structures to those of *E.alarconi* sp. nov.

Another interesting morphological trait apparently differing between species of the genus *Elpidium*, according to the literature, is the strength of the separation between segments 4a and 4b of the antennula. Most species have these segments only partially or weakly separated, as described for *E.maricaoense*, *E.littlei*, *E.wolfi*, *E.litoreum*, *E.cordiforme*, *E.laesslei*, *E.merendonense*, *E.heberti*, *E.oxumae*, *E.picinguabaense*, *E.eriocaularum*, and *E.higutiae* (Tressler 1941; Pinto and Jocqué 2013; Pereira et al. 2019, 2022, 2023), while others, including *E.bromeliarum*, *E.martensi*, and *E.purium* are described as having a single, undivided, fourth segment (Pinto and Purper 1970; Danielopol et al. 2014; Pereira et al. 2023). Consequently, most authors considered a five-segmented antennula as a diagnostic character of the genus (Pinto and Jocqué 2013; Danielopol et al. 2014; Pereira et al. 2019). However, *E.alarconi* sp. nov. shows a distinctly clear separation between segments 4a and 4b in most specimens (weaker in others) under standard microscopic observation in transmitted light, so that its antennula appears as having six segments, rather than five. Also six segments are apparent in the graphic description of *E.heberti*; although the authors indicate that the fourth segment is “partially subdivided” when describing the species in the text, it is drawn as divided with a continuous line in their figure (Pereira et al. 2019: fig. 9a), while other species described in the same publication show a dashed line. In the original description of the type species, and in a subsequent revision and establishment of neotypes, Müller (1881) and Pinto and Purper (1970) stated that the antennula usually has five segments, but that it can exceptionally have six. Pinto and Jocqué (2013) suggested this separation might not be fully functional. Later, Pereira et al. (2022), when performing a phylogenetic analysis of the genus using a list of coded characters (detailed in the Supplementary information of their publication), characterized all species for which they found information on this trait, as having a partially fused fourth segment of the antennula. They concluded, after re-examining preparations of most species, that this morphological feature was shared for all *Elpidium* species analyzed, and considered that, according to microscopic observations, the segmentation was most probably only occurring on one side of the segment, but not in the other (Pereira, pers. comm.). We tested this possibility in the case of *E.alarconi* sp. nov., and could confirm it; even if the separation was quite clear under standard transmitted light, the use of fluorescence and scanning microscopy confirmed that it was partial, and only present in the inner side of the segment for each antennula. It remains to be confirmed whether this feature is shared with all other species of the genus, and which is its functional and evolutionary significance.

**Figure 9.**
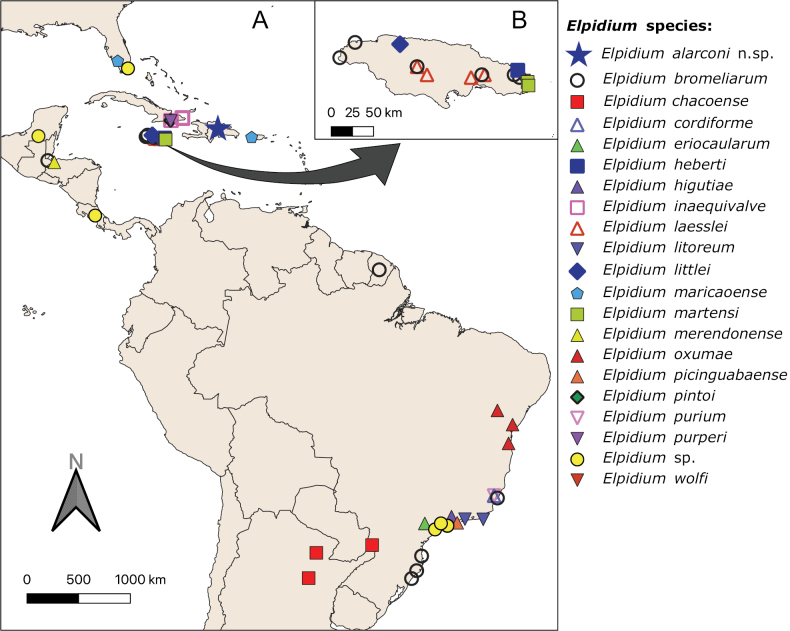
World distribution map (**A**) of *Elpidium* records, according to published information **B** detail of distribution in Jamaica. Note that some records of *E.bromeliarum* are considered doubtful (see text for further explanation).

### ﻿Diversity and biogeography

The species described in this work represents the first member of the genus *Elpidium* identified to species level for the island of Hispaniola; it must be noticed that Acosta-Mercado et al. (2012) previously recorded two undetermined species collected from liverworts. Its presence in this island does not come as a surprise, considering that several *Elpidium* species had been found in the neighboring islands of Cuba, Jamaica, and Puerto Rico, in addition to those found in the mainland (Fig. 9). At present, Jamaica can be considered the area with the highest density of *Elpidium* species worldwide, most of them endemic to the island (Little and Hebert 1996; Danielopol et al. 2014; Pereira et al. 2019). The high diversity and endemicity of the genus *Elpidium* was previously noted by Little and Hebert (1996), based on allozyme and mitochondrial data and on partial description of hemipenis morphologies, although they did not formally describe any species. They also highlighted the role of isolation and restricted dispersal in phytotelma ostracods for speciation, although more recent works have shown how they may disperse via phoresis using mostly amphibians and snakes (Lopez et al. 1999, 2005; Sabagh and Rocha 2014; Cunha et al. 2023). The high diversity and endemicity of *Elpidium* has been further supported by studies in Brazil and Argentina during the past few years (Pereira et al. 2022, 2023; Díaz et al. 2024), and corroborated by the present survey. It seems therefore that the species diversity of the genus *Elpidium* may be much higher than previously expected. The small number of samples collected hitherto from phytotelmata in tropical countries probably caused that only 20 species of *Elpidium* are known to date, but we expect many more to be discovered in the future, considering the large areas in the Neotropics that have remained unexplored for this habitat (Jocque et al. 2013; Pereira et al. 2022) (Fig. 9).

The high endemicity of *Elpidium* species is challenged by the wide distribution of the type species *E.bromeliarum*, recorded from Southern Brazil to Central America and Jamaica (Fig. 9). However, we must be cautious, as probably most of the records out of Brazil might be erroneous (Pereira et al. 2023). Indeed, even if some authors cited *E.bromeliarum* from Costa Rica (Pinto and Jocqué 2013; Pereira et al. 2023) based on early work by Picado (1913), this author did not confirm that the *Elpidium* species he found was *E.bromeliarum*, but a similar species: “Metacypris (Elpidium) sp. (fig. 42, B). La Mica, 1500 mètres. Ce crustacé est, d’après, G. W. Müller une espèce très voisine d’*Elpidiumbromeliarum*. Quand le Crustacé est vivant, il présente cependant une pigmentation différente de celle de l’espèce décrite par Fritz Müller...” (Picado 1913: 336). [Transl: “Metacypris (Elpidium) sp. (fig. 42, B). La Mica, 1500 metres. This crustacean is, according to G. W. Müller, a species closely related to *Elpidiumbromeliarum*. When the crustacean is alive, however, it presents a different pigmentation from that of the species described by Fritz Müller...”]. Picado (1913) included *Elpidiumbromeliarum* in his list of “Animaux bromelicoles actuellement connus”, but he specifically wrote that this species lived in Brazilian epiphytic bromeliads, not in Costa Rica. Later on, it was Tressler (1956) who recorded *E.bromeliarum* from Jamaica (Fig. 9), although he did not discuss or show diagnostic characters of the hemipenis, so it may be considered an unreliable record (Pereira et al. 2023). The presence of *E.bromeliarum* in Guatemala (Pérez et al. 2012) should also be considered doubtful, because the authors only provided valve pictures, and it would be necessary to check the morphology of the copulatory apparatus to confirm this determination. Furthermore, Pinto and Jocqué (2013) described *E.merendonense* one year later from Honduras; it would therefore be interesting to check whether or not the species determined as *E.bromeliarum* from Guatemala may actually belong to a different species, perhaps *E.merendonense*. Finally, *E.bromeliarum* has also been recorded from French Guiana (GBIF.Org 2023), but we could not find further information on morphological aspects of this record, which is quite far from other geographic locations of the species, so we consider it should be taken with caution. Actually, the confusion on the identification and distribution of *E.bromeliarum* can be traced back to its discovery; in his description of male copulatory organs, Müller (1881) included at least three different morphologies of the hemipenis distal lobe, suggesting it was very variable. However, these different morphotypes most probably belong to different species of *Elpidium*. This confusion was continued in the review of Pinto and Purper (1970), as they also showed some clearly different hemipenes as belonging to the same species, although they may actually correspond to different ones (Pereira et al. 2017, 2023).

Another potential issue for understanding the biogeography of *Elpidium* is the presence of *E.maricaoense* in Florida (Tressler 1956). Even though this record was noted by the same author who described the species earlier from Puerto Rico (Tressler 1941), and considering the high diversity of species in the Caribbean and the lack of morphological information for the Florida specimens, the presence of *E.maricaoense* in mainland America needs to be corroborated by further sampling in Florida. In addition, undetermined species of *Elpidium* have been recorded from other locations besides Costa Rica (Picado 1913), including Brazil, Florida and Mexico (Mercado-Salas et al. 2021; GBIF.Org 2023), so we would expect the genus to be widespread in the Neotropical region, and many more species to be described in the future. Consequently, the early suggestion by Müller (1881) that *E.bromeliarum* should be widely distributed in Brazil, is not corroborated by recent data, although it has been shown that the genus *Elpidium* has probably colonised most of the Neotropical region.

The new finding of *E.alarconi* sp. nov. in Hispaniola should initially be considered as an endemism for the island. However, considering that it was collected from bromeliads in managed gardens or nearby secondary forests, it would not be surprising that future research may record it in other regions, considering also its morphological proximity to several mainland species, and the worldwide proliferation of exotic ostracods driven by human movements (McKenzie and Moroni 1986; Valls et al. 2014). This might be one of the reasons for the lack of congruence between the geographic distribution of *Elpidium* species and their phylogenetic relationship using morphological data (Pereira et al. 2022). These authors only found a clear relationship between a clade of *Elpidium* species and their restricted distribution in Jamaica. They suggested that the lack of a phylogeographic pattern for most of the species relies on the scarcity of studies and/or the lack of critical morphological information for some species described long ago. We agree that these are the main reasons for the unresolved *Elpidium* biogeography, although we would not discard human-mediated movement of *Elpidium* species through bromeliad trade for gardening, as shown for other ostracod species in relation to the trade of aquatic plants for cultivation, gardening or aquaculture (McKenzie and Moroni 1986; Matzke-Karasz et al. 2014; Valls et al. 2014; Smith et al. 2024). Pereira et al. (2023) proposed using the genus *Elpidium* as a model group to study biogeographic areas of endemism, but considering the issue of expanding exotic ostracods, this kind of studies should be focused on sampling bromeliads mostly in undisturbed environments, far from human-impacted sites.

### ﻿Phylogeny and systematics

Our molecular phylogeny analysis placed *E.alarconi* in the same clade as *Metacypris*, *Cytheridella* and *Gomphodella*, and far from the branch where *Limnocythere* was positioned in the phylogenetic tree. Assuming that the *Limnocythere* specimen whose DNA sequence is deposited in the repository has been accurately identified, and that it is representative of the Limnocytherinae, these results provide further support for the suggestion that the former subfamilies Timiriaseviinae and Limnocytherinae should be promoted to family level (Tanaka et al. 2021). The Timiriaseviinae subfamily was established to accommodate a fossil species of the genus *Timiriasevia* by Mandelstam (in Kashevarova et al. 1960). Shortly after, the subfamily Metacyprinae was established by Danielopol (1965), initially as a tribe (Metacyprini) of the subfamily Limnocytherinae, to include the genera *Metacypris*, *Elpidium*, *Afrocythere* and *Cordocythere*. Later on, the tribe Metacyprini was promoted to subfamily, and considered a junior synonym of the Timiriaseviinae (Colin and Danielopol 1978; Danielopol et al. 2018).

Although most recent authors consider the Timiriaseviinae a subfamily included in the Limnocytheridae Sars, 1928 together with the subfamily Limnocytherinae, after considering their major differences in shell and soft parts anatomy (Martens 1995; Danielopol et al. 2018) and the long genetic distance between them (Tanaka et al. 2021), we decided to accept the proposal of these last authors to promote the Timiriaseviinae to family Timiriaseviidae, as also did earlier Mesquita-Joanes et al. (2024), although the molecular basis for this promotion needs to be further tested with more sequences of species belonging to the Limnocytheridae s.s. Nevertheless, despite some morphological similarities between both families, which might be considered large enough as to hamper the proposed change of taxonomical levels suggested by Tanaka et al. (2021) and adopted here, we consider that the differences between them are even stronger, supporting a separation in two distinct families. Regarding similarities, there are three characters that are shared between both groups (Danielopol et al. 2018), but which can be considered relatively weak or even plesiomorphic, and therefore not well founded for their use in sustaining their monophyly: i) the distal antennular aesthetasc, fused with a distal seta, shows a much longer fused zone in the Limnocytheridae s.s. than in the Timiriaseviidae, in which this fusion is very short (as in *Gomphodella* or *Gomphocythere*) or even not observed (in *Elpidium*, *Intrepidocythere*, or *Metacypris*); ii) the presence of three claws in the last segment of the antenna may be regarded as an important trait, but it might be considered plesiomorphic, as it appears also in the primitive Bythocytheridae, and Entocytheridae; and iii) the presence of a minute seta in the last podomere of thoracopods, this segment fused with the final claw, may be a remnant of the posterior seta that some other Cytherocopina hold in the last segment of thoracopods (when it is not fused with the claw). For instance, it is the only posterior seta present in the thoracopod endopods of some Cytherocopina (e.g., in *Terrestricythere*, *Bythocypris*, *Bairdoppilata*) or in Darwinuloidea (e.g., in *Vestalenula*). It is interesting to notice how the first thoracopod of adult males of *Terrestricythere* hold a small posterior seta in their modified claw, probably resulting from the fusion of the last segment with the claw, as the male endopod has only two segments, while there are three in the female (Horne et al. 2004). Furthermore, we can see a similar shape of a fused segment-claw with a tiny seta in *Amnicythereprespensis* (in Petkovski and Keyser 1992), a species in the family Leptocytheridae, therefore also outside the Limnocytheridae s.l. Conversely, there are some species of Limnocytheridae s.l. for which that minute seta has not been observed or illustrated, as it occurs in several *Limnocythere* (Martens, 1990) or in *Intrepidocythere* (Pinto et al. 2008), although it may have been missed by the authors when illustrating them. Consequently, it does not seem appropriate to keep Timiriaseviinae and Limnocytherinae together in the same family on the basis of such a loose character of a minute seta, considering that it is not present in all species, and that it is also present in other species outside the family Limnocytheridae s.l., suggesting it is a plesiomorphic trait.

Regarding morphological differences between Limnocytheridae s.s. and Timiriaseviidae, we consider these are more consistent and strong enough as to support their separation as two distinct families: (i) unlike the Timiriaseviidae, females of the Limnocytheridae s.s. do not have a brooding chamber in their valves. This is an important morphological trait, related to reproduction and readily observed in the female carapace of most Timiriaseviidae; (ii) another very important trait, in our view, is the segmentation of the maxillular palp. It has only one segment in the Timiriaseviidae (*Elpidium*, *Cytheridella*, *Intrepidocythere*, *Metacypris*, *Gomphodella*, *Gomphocythere*) but two in the Limnocytheridae s.s. (e.g., in *Limnocythere*, *Korannacythere* and *Leucocythere*); (iii) still another important trait differing between the two families is the ventral seta on the second antennular segment, which is situated in a medial or proximal position in the Timiriaseviidae, but in the distal margin in the Limnocytheridae; in addition, (iv) the antennula is five-segmented in the Limnocytheridae, but in the Timiriaseviidae it can be five-segmented (as in *Cytheridella*), six-segmented (as in *Metacypris* and *Gomphocythere*) or with a partial segmentation of the 4^th^ segment, i.e. apparently six-segmented but not completely (as in *Elpidium*, *Gomphodella* or *Intrepidocythere*) and (v) the distal lobe of the hemipenis is articulated in the Timiriaseviidae, but not in the Limnocytheridae. This can be considered an important trait as well, because of its potential functional role in reproduction. Furthermore, (vi) a recent review of the sieve-type pore canals (StPC) in the Limnocytheridae s.l. by Danielopol et al. (2018) concluded that these pore canals, when present, have a seta inside them in the Limnocytheridae s.s. (type C StPC) but not in the Timiriaseviidae (type B StPC). Finally, if this taxonomic scheme with two separate families (Limnocytheridae s.s. and Timiriaseviidae) is accepted, a derived conclusion should be to promote their constitutive tribes to subfamilies: Timiriaseviini Mandelstam, 1960, Cytheridellini Danielopol & Martens, 1989 and GomphodelliniDanielopol et al., 2018 would therefore change to Timiriaseviinae Mandelstam, 1960, Cytheridellinae Danielopol & Martens, 1989 and GomphodellinaeDanielopol et al., 2018, all belonging to the family Timiriaseviidae; and the Limnocytheridae s.s. would be composed by the subfamilies Leucocytherinae Danielopol & Martens, 1989 and Limnocytherinae Klie, 1938 (previously as tribes Leucocytherini Danielopol & Martens, 1989 and Limnocytherini Klie, 1938, belonging to the subfamily Limnocytherinae Sars, 1928).

Within the Timiriaseviidae, previous phylogenies using morphological traits, positioned the genus *Elpidium* either alone in a branch separated from another that included *Gomphodella*, *Metacypris* and *Cytheridella* (Karanovic 2009) or together with *Metacypris* in a branch separated from *Gomphodella* or *Cytheridella* (Karanovic and Humphreys 2014). In contrast, our 18S phylogenetic tree suggests *Elpidium* might be closer to *Gomphodella* than to *Metacypris* or *Cytheridella*. Such different pattern has consequences for the interpretation of the biogeographic origin of *Elpidium*; as *Gomphodella* is exclusive to the Australian region, the phylogenetic association between these two genera suggests an ancient vicariant origin from the breakage of Gondwana, when Australia became separated from Antarctica and South America, similar to the findings of Sigvardt et al. (2021) for *Lynceus* (Laevicaudata). Therefore, our findings do not support the alternative process of a dispersal event from Eurasia or Africa to South America, as previously proposed by Karanovic (2009) in relation to the morphological similarities between *Elpidium* and *Metacypris*, and to the rich fossil record of the latter. Yet some morphological traits point to other relationships. For instance *Elpidium* lack StPC, while *Gomphodella* or *Cytheridella* present this type of pores on its valves (Danielopol et al. 2018). Another interesting trait is the row of posteroventral type-A2 pores (with rim and seta) on the peripheral marginal infold of valves of *Elpidium*. A similar row of pores is observed in *Cytheridella* (Danielopol et al. 2023: fig. 11) and in *Intrepidocythereibipora* (Pinto et al. 2008), although in a more external position in this case. Still another particular trait shared by *Elpidium*, *Intrepidocythere* and *Cytheridella* is the presence of a seta at the base of the articulated distal lobe of hemipenes, not described in other species of the family. The inconsistencies between morphological and molecular phylogenetic relationships inside the family calls for further molecular analysis of other genera of Timiriaseviidae, and a more detailed morphological work, which combined would help understanding the phylogeny and early biogeography of this interesting family of non-marine ostracods.

## ﻿Conclusions

With the description of a new species of *Elpidium* from Hispaniola, we fill the gap of the only island of the Greater Antilles for which no determined species of the genus were known to occur hitherto. *Elpidiumalarconi* sp. nov. has a shell morphology similar to other species of the genus (e.g., with valves covered with minute foveolae, posteriorly truncated in dorsal view), but the distinct shape of its hemipenis distal lobe and lower ramus separates it from other *Elpidium* species. Pereira et al. (2022) classified *Elpidium* species in two groups: those with the hemipenis copulatory process divided, and those with it undivided, to which the new species belong. The first group is restricted to Jamaica, but the second has an inconsistent phylogeographic pattern. As these authors suggest, we need a higher coverage of taxonomic and biogeographic information of the genus in the Neotropics to be able to better understand the phylogeny and biogeographic history of *Elpidium* ostracods. At a wider phylogenetic context, the available molecular data show how *Elpidium* is tightly related to *Gomphodella*, *Metacypris* and *Cytheridella*, but not to *Limnocythere*, supporting the establishment of the Timiriaseviidae as a family (Tanaka et al. 2021), not as a subfamily as previously considered. Despite the still reduced molecular information, which should be increased in the future to evaluate if the pattern holds when including more sequences of other limnocytherid species, we consider that there is already a suficient number of large differences in key morphological traits that further support the promotion of the subfamily Timiriaseviinae to a family level, separated from the Limnocytheridae s.s., such as the brood space in Timiriaseviidae female carapaces, or the articulated distal lobe in male hemipenes, among others.

## Supplementary Material

XML Treatment for Timiriaseviidae

XML Treatment for Elpidium

XML Treatment for Elpidium alarconi

## ﻿Additional information

### Author ORCIDs

Francesc Mesquita-Joanes https://orcid.org/0000-0001-7168-1980

Ángel Gálvez https://orcid.org/0000-0002-5562-7316

Ferran Palero https://orcid.org/0000-0002-0343-8329

Juan Rueda https://orcid.org/0000-0002-7629-8881

### Data availability

All of the data that support the findings of this study are available in the main text.
